# Vascular Positron Emission Tomography and Restenosis in Symptomatic Peripheral Arterial Disease: A Prospective Clinical Study

**DOI:** 10.1016/j.jcmg.2019.03.031

**Published:** 2020-04

**Authors:** Mohammed M. Chowdhury, Jason M. Tarkin, Mazen S. Albaghdadi, Nicholas R. Evans, Elizabeth P.V. Le, Thomas B. Berrett, Umar Sadat, Francis R. Joshi, Elizabeth A. Warburton, John R. Buscombe, Paul D. Hayes, Marc R. Dweck, David E. Newby, James H.F. Rudd, Patrick A. Coughlin

**Affiliations:** a Division of Vascular Surgery, Department of Surgery, Addenbrooke’s Hospital, University of Cambridge, United Kingdom; b Department of Cardiovascular Medicine, Addenbrooke’s Hospital, University of Cambridge, United Kingdom; c Cardiovascular Research Center, Division of Cardiology, Massachusetts General Hospital, Harvard Medical School, Boston, Massachusetts; d Department of Clinical Neurosciences, University of Cambridge, United Kingdom; e Statistical Laboratory, Department of Pure Mathematics and Mathematical Sciences, University of Cambridge, United Kingdom; f Heart Center, Rigshospitalet, Denmark; g Department of Nuclear Medicine, Addenbrooke’s Hospital, University of Cambridge United Kingdom; h British Heart Foundation for Cardiovascular Science, University of Edinburgh, Edinburgh, United Kingdom

**Keywords:** atherosclerosis, computed tomography, ^18^F-fluorodeoxyglucose, ^18^F-sodium fluoride, peripheral arterial disease, positron emission tomography, restenosis, AU, Agatston unit, CI, confidence interval, CLI, critical limb ischemia, CT, computed tomography, DCB, drug-coated balloon, FDG, fluorodeoxyglucose, hsCRP, high-sensitivity C-reactive protein, IQR, interquartile range, NaF, sodium fluoride, PAD, peripheral arterial disease, PET, positron emission tomography, PTA, percutaneous transluminal angioplasty, SFA, superficial femoral artery superficial femoral artery, SUV, standardized uptake value, TBR, target-to-background ratio

## Abstract

**Objectives:**

This study determined whether *in vivo* positron emission tomography (PET) of arterial inflammation (^18^F-fluorodeoxyglucose [^18^F-FDG]) or microcalcification (^18^F-sodium fluoride [^18^F-NaF]) could predict restenosis following PTA.

**Background:**

Restenosis following lower limb percutaneous transluminal angioplasty (PTA) is common, unpredictable, and challenging to treat. Currently, it is impossible to predict which patient will suffer from restenosis following angioplasty.

**Methods:**

In this prospective observational cohort study, 50 patients with symptomatic peripheral arterial disease underwent ^18^F-FDG and ^18^F-NaF PET/computed tomography (CT) imaging of the superficial femoral artery before and 6 weeks after angioplasty. The primary outcome was arterial restenosis at 12 months.

**Results:**

Forty subjects completed the study protocol with 14 patients (35%) reaching the primary outcome of restenosis. The baseline activities of femoral arterial inflammation (^18^F-FDG tissue-to-background ratio [TBR] 2.43 [interquartile range (IQR): 2.29 to 2.61] vs*.* 1.63 [IQR: 1.52 to 1.78]; p < 0.001) and microcalcification (^18^F-NaF TBR 2.61 [IQR: 2.50 to 2.77] vs*.* 1.69 [IQR: 1.54 to 1.77]; p < 0.001) were higher in patients who developed restenosis. The predictive value of both ^18^F-FDG (cut-off TBR_max_ value of 1.98) and ^18^F-NaF (cut-off TBR_max_ value of 2.11) uptake demonstrated excellent discrimination in predicting 1-year restenosis (Kaplan Meier estimator, log-rank p < 0.001).

**Conclusions:**

Baseline and persistent femoral arterial inflammation and micro-calcification are associated with restenosis following lower limb PTA. For the first time, we describe a method of identifying complex metabolically active plaques and patients at risk of restenosis that has the potential to select patients for intervention and to serve as a biomarker to test novel interventions to prevent restenosis.

Lower limb peripheral arterial disease (PAD) is the third leading cause of atherosclerotic cardiovascular morbidity, following coronary artery disease and stroke (1). PAD is a global problem, affecting more than 200 million people worldwide, and is present in 20% of the population older than the age of 75 years (2). Although 10% of patients with PAD are asymptomatic (2), intermittent claudication is the most common symptom with more severe limb ischemia causing rest pain, ulceration, and gangrene. Without treatment, patients with severe limb ischemia are at high risk of lower limb amputation.

Revascularization is the mainstay of treatment for symptomatic lower limb PAD. The endovascular route offers several advantages over open surgical revascularization and is increasingly preferred. Percutaneous transluminal angioplasty (PTA) of the superficial femoral artery (SFA) is the most common endovascular intervention performed, but restenosis rates of 40% to 60% at 12 months limit its long-term durability 3, 4. Arterial restenosis after PTA is a difficult problem to solve, requiring further attempts at revascularization with associated poorer clinical outcomes and increased cost (5). The mechanisms underlying vascular restenosis are complex but may include inflammation and calcification (6). Currently, we are unable to predict which patients will experience restenosis.

Positron emission tomography (PET) is a clinically available molecular imaging method that produces quantitative images of the distribution of a radiolabelled molecule. Such images can be co-registered with computed tomography (CT) to provide a topographical distribution of activity. ^18^F-Fluorodeoxyglucose (^18^F-FDG), a radio-labelled glucose analogue, is taken up by all glucose-metabolizing cells. ^18^F-FDG PET/CT is widely used in oncology to diagnose primary and metastatic cancer. The technique has been adapted to measure arterial inflammation in several vascular beds. Excessive ^18^F-FDG uptake is associated with increased vascular macrophage burden and future cardiovascular events and is amenable to modulation by statins, anti-inflammatory therapies, and therapeutic lifestyle changes.^18^F-Sodium fluoride (^18^F-NaF) is a bone PET tracer, first introduced in 1962 for the detection of osteogenic activity (7). More recently, ^18^F-NaF has been shown to localize to developing microcalcifications, providing a marker of calcification activity that has been investigated in a range of cardiovascular disorders including aortic stenosis, carotid and coronary atherosclerosis, and abdominal aortic aneurysm disease 8, 9.

The aim of this prospective imaging study was to use PET/CT imaging to investigate the contribution of inflammation (with ^18^F-FDG) and calcification activity (with ^18^F-NaF) to the development of arterial restenosis after SFA balloon angioplasty, in patients with symptomatic PAD. PET/CT imaging was performed before angioplasty and then repeated 6 weeks after the procedure to assess whether either baseline or post-procedure tracer uptake might predict those at risk of restenosis.

## Methods

### Patients

**Figure 1:**
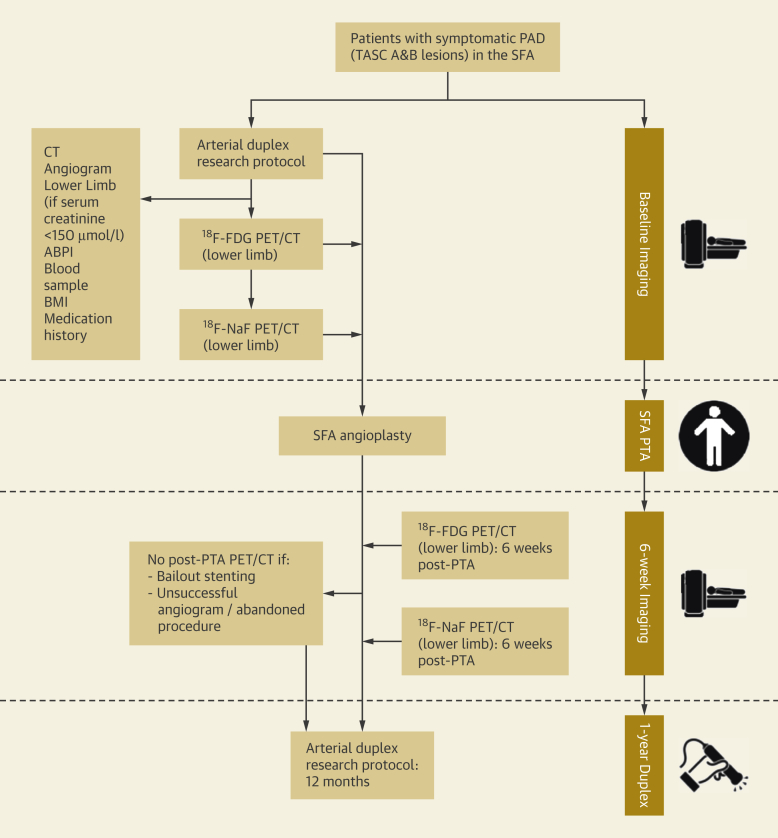
The CIRLA Study Protocol Study protocol flow stream illustrated on the left, with accompanying timeline on the right. ABPI = ankle-brachial pressure index; BMI = body mass index; CIRLA = Calcification and Inflammation on Restenosis rates following Lower limb Angioplasty; ^18^F-FDG = ^18^F fluorodeoxyglucose; ^18^F-NaF = ^18^F-sodium fluoride; PAD = peripheral arterial disease; PET/CT = positron emission tomography-computed tomography; PTA = percutaneous transluminal angioplasty; SFA = superficial femoral artery; TASC = TransAtlantic Inter-Society Consensus.

Patients with symptomatic PAD who were older than the age of 55 years were recruited from Addenbrooke’s Hospital, Cambridge, United Kingdom. Subjects had unilateral symptoms of either intermittent claudication or critical limb ischemia (CLI) and a planned SFA angioplasty (Figure 1). The SFA lesion classification was either type A or B (based on TransAtlantic Inter-Society Consensus II classification system [TASC]) (10). Exclusion criteria were as follows: renal impairment (estimated glomerular filtration rate <30 ml/min/1.73 m^2^), diabetes mellitus requiring insulin therapy, a history of contrast nephropathy (previously documented absolute or relative increase in serum creatinine at 48 to 72 h after exposure to contrast agent [11]) or allergy to iodinated contrast, presence of metastatic malignancy, inability to provide written informed consent, inability to lie flat during imaging, ipsilateral SFA angioplasty within the previous 6 months, and patients undergoing concomitant adjunct endovascular treatment (with bare-metal or drug-eluting stents).

The baseline clinical evaluation included assessment of cardiovascular risk factors, measurement of bilateral ankle-brachial pressure indices, and clinical biochemistry for lipid, calcium, creatinine, and high-sensitivity C-reactive protein (hsCRP) concentrations (Figure 1). All patients provided written informed consent. The study was approved by the local research ethics committee (Cambridge East Research Ethics Committee, Reference 15/EE/0153), and was performed in accordance with the Declaration of Helsinki. A license to administer radioactive medicinal products to consenting participants was granted by the UK Administration of Radioactive Substances Advisory Committee (RPC 83/400/33632). This study is registered as ISRCTN34690731.

### Index lesion

The index lesion was defined as the SFA atherosclerotic lesion treated with PTA and was defined anatomically based on previous methods (Supplemental Figure 1) 12, 13, 14. The anatomic boundaries of the index lesion were defined using both the arterial duplex and CT scan at the site of luminal stenosis and subsequent treatment. Angioplasty was performed in accordance with standard practice for lower limb intervention (15). In cases of multiple lesions (TASC B), the index lesion comprised all segments with a luminal stenosis (as per TASC II classification) (16), the length of which was measured using both duplex and CT. Consequent analysis of PET/CT, CT calcium score, and restenosis based on duplex scanning pertains to this area, the ‘index lesion’.

### Procedures

We used a combination of non-invasive imaging techniques. Imaging data were collected from both ^18^F-FDG and ^18^F-NaF PET/CT scans, CT angiograms, unenhanced CT scans, and arterial duplex images (Figure 1).

^18^F-FDG and ^18^F-NaF PET scans were performed using validated methods 9, 17 on a GE Discovery 690 combined PET/CT system (GE Healthcare, Little Chalfont, United Kingdom). Patients underwent both ^18^F-FDG and ^18^F-NaF PET scans before PTA and these were repeated 6 weeks after angioplasty. Each pair of ^18^F-FDG and ^18^F-NaF PET scans was acquired within 7 days of each other. Images were reconstructed using a standard clinical VUE-point FX time-of-flight algorithm. Image analysis was performed on an Apple Macintosh computer (Apple Inc., Cupertino, California) using an open-source DICOM viewer (version 9.0.1, OsiriX MD Imaging Software, Pixmeo, Geneva, Switzerland). SFA ^18^F-FDG and ^18^F-NaF uptake values were quantified, as described below. Briefly, from the acquired raw data, the scans were reconstructed into 3-mm slices. Circular regions of interest were placed around the SFA on adjacent axial images. The maximum standardized uptake value (SUV) for each identified slice was recorded. Target-to-background ratio (TBR) was calculated by dividing the maximum arterial SUV by blood pool activity (the mean SUV from 5 circular regions of interest drawn in the center of the common femoral vein). The arterial slices that spanned the length of the index lesion were then used to calculate the index lesion TBR_max_.

The CT calcium score of the index lesion was measured on non-contrast CT images of the SFA using the freely available ‘Calcium Scoring’ plug-in for the OsiriX software. The method of Agatston et al. (18), based on an attenuation threshold of 130 Hounsfield units in 3 contiguous voxels, was used (19). The extent of calcification was expressed as a score in Agatston units (AU).

The primary outcome was restenosis of the angioplastied index lesion, defined as a SFA stenosis of ≥50% (20), measured during a repeat study arterial duplex scan performed 12 months after angioplasty (as part of the study protocol). The full length of the SFA (defined anatomically) was analyzed in 4-cm lengths in keeping with an ultrasound probe length of 40 mm. Time to restenosis was also determined in those patients who re-presented to the vascular surgery department with symptom recurrence and a duplex-defined restenosis before the end of the 12-month follow-up period.

### Statistical analysis, reproducibility, and power calculation

All statistical analysis was performed using SPSS (version 25, IBM, Armonk, New York) and The R Project for Statistical Computing Programme (version 3.5.1, Vienna, Austria). Continuous variables were expressed as mean ± SD for normally distributed data and median (interquartile range [IQR]) for skewed distributions. Parametric (unpaired and paired *t*-tests) and nonparametric (Mann-Whitney *U*) tests were used for normally distributed and skewed data, respectively. After appropriate checks for normality, TBR_max_ values for patients with and without restenosis were analyzed using the Mann-Whitney *U* test. Baseline correlations between ^18^F-FDG, ^18^F-NaF, and risk factors were examined using Spearman rank correlation coefficients (nonparametric data). Intra-class correlation coefficients were calculated to assess average intra-observer (1-way random effects model) and inter-observer (2-way random effects model) agreement of TBR_max_ measurements. Kolmogorov-Smirnoff tests were used to compare the distribution of PET tracer uptake values for patients with restenosis to the distribution for patients without, and Kaplan-Meier survival analyses were used to determine restenosis probabilities. Cut-off values were determined using R function rpart to fit classification tree data. Data are presented as n (%), mean ± SD or 95% confidence interval (CI), or median (interquartile range) as appropriate. Two-sided p values were used in all cases, with values <0.05 considered to be significant.

See the Supplemental Appendix for further statistical methodology.

## Results

A total of 86 patients were identified as eligible for enrollment. Fifty-five patients were eligible for inclusion and 50 patients agreed to participate (Supplemental Table 1). Ten patients did not complete the study protocol because of the following: stenting performed due to a suboptimal PTA (n = 5), patient withdrawal from the study before the 6-week scan (n = 3), and no angioplasty performed because of an unfavorable lesion profile at the time of angioplasty (n = 2). Subsequent results, therefore, refer to the 40 patients who successfully completed the study protocol (Table 1), with no baseline differences between the restenosis and no-restenosis cohorts. Characterization of PAD symptoms was consistent with the diagnosis of CLI as Rutherford Grade 3, Category 5 (Table 1) (21).

**Table 1:** Clinical Characteristics of Patients Stratified by Restenosis Status (N = 40)

	All (N = 40)	No Restenosis (n = 26)	Restenosis (n = 14)	p Value
Age in yrs,	71.5 (64.8–79.3)	73.5 (67.5–80.5)	63 (60–75)	0.051
Men	26 (65)	17 (65)	9 (64)	0.945
Subgroup				
Intermittent claudication	31 (78)	19 (73)	12 (86)	0.367
Critical limb ischemia	9 (23)	7 (27)	2 (14)	0.125
TASC A	26 (65)	19 (73)	7 (50)	0.243
TASC B	14 (35)	7 (27)	7 (50)	0.327
Previous medical history				
Hypertension	32 (80)	19 (73)	13 (93)	0.141
Non-insulin dependent diabetes	14 (35)	9 (35)	5 (36)	0.945
Ischemic heart disease/MI	15 (38)	7 (27)	8 (57)	0.063
Cerebrovascular event/TIA	5 (13)	3 (12)	2 (14)	0.805
Smoker	4 (10)	3 (12)	1 (7)	0.663
Ex-smoker	36 (90)	22 (85)	13 (93)	0.572
Medication				
Clopidogrel	6 (15)	5 (19)	1 (7)	0.178
Aspirin	26 (65)	15 (58)	11 (79)	0.652
Anticoagulation	4 (10)	3 (12)	1 (7)	0.892
Dipyridamole	1 (3)	1 (4)	-	0.546
Statin	36 (90)	22 (85)	13 (93)	0.458
ACE inhibitor	20 (50)	13 (50)	7 (50)	0.982
BMI (kg/m^2^)	28.57 ± 4.35	28.84 ± 4.84	28.07 ± 3.37	0.821
ABPI	0.72 (0.68–0.77)	0.74 (0.68–0.79)	0.70 (0.66–0.75)	0.154
Lipid profile (mmol/l)				
Total cholesterol	4.38 ± 1.05	4.40 ± 1.08	4.33 ± 1.04	0.777
HDL cholesterol	1.27 ± 0.36	1.29 ± 0.40	1.23 ± 0.28	0.766
LDL cholesterol	2.17 ± 0.84	2.23 ± 0.75	2.06 ± 1.00	0.328
Triglycerides	2.01 ± 0.96	1.98 ± 1.05	2.29 ± 0.77	0.201
HDL:cholesterol	3.68 ± 1.14	3.7 ± 1.21	3.65 ± 1.02	0.989
Serum calcium and creatinine				
Creatinine (μmol/l)	78.5 (67.75–89.25)	78.5 (65.25–89.75)	79 (72–88.75)	0.766
Corrected calcium (mmol/l)	2.36 (2.31–2.42)	2.33 (2.31–2.42)	2.38 (2.35–2.41)	0.348
High sensitivity CRP (mg/dl)	2.77 (1.10–8.80)	3.90 (1.19–8.71)	2.1 (1.05–8.61)	0.61

Values are median (interquartile range), n (%), or mean ± SD.

ABPI = ankle-brachial pressure index; ACE = angiotensin-converting enzyme; CRP = C-reactive protein; HDL = high-density lipoprotein; LDL = low-density lipoprotein; MI = myocardial infarction; TASC = TransAtlantic Inter-Society Consensus; TIA = transient ischemic attack.

The median time from angioplasty to the study exit ultrasound scan was 371 (IQR: 365 to 385) days. Fourteen patients experienced the primary outcome of development of anatomic restenosis, with the median time to identification of restenosis being 179 (IQR: 126 to 231) days.

### PET imaging

The median time from baseline PET/CT imaging (Central Illustration) to index lesion angioplasty was 21 (IQR: 12 to 38) days. The median time from successful angioplasty to follow-up PET/CT imaging was 46 (IQR: 43 to 48) days.

**Central Illustration:**
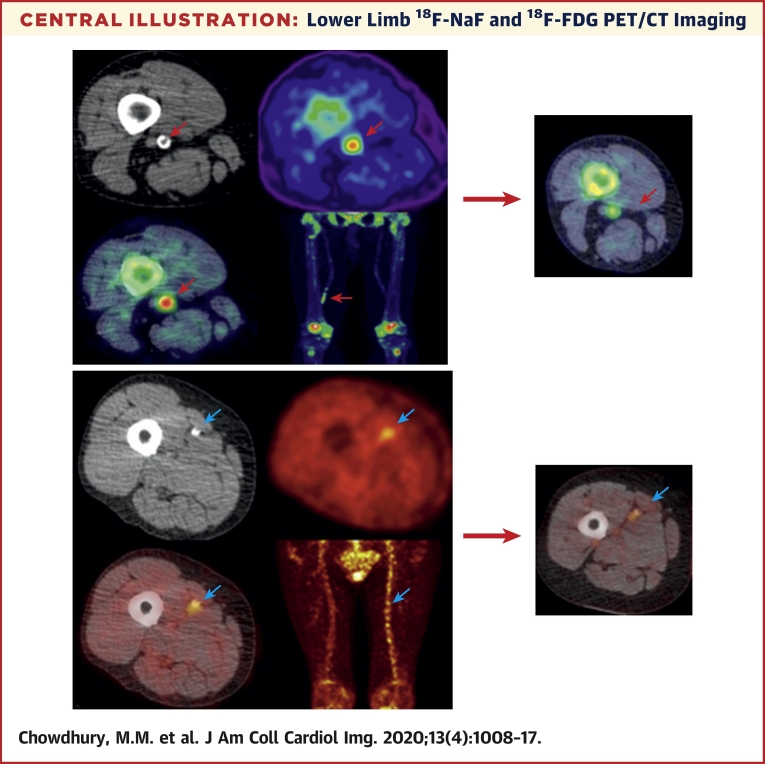
Lower Limb ^18^F-NaF and ^18^F-FDG PET/CT Imaging Non-contrast CT **(top left)** with a rim of calcification of the vessel, ^18^F-NaF PET **(top right)**, and fused ^18^F-NaF PET/CT **(bottom left)** of the superficial femoral artery **(red arrow)** at the level of the adductor canal, demonstrating significant vessel uptake in patient with claudication at 300 yards. In addition, there is prominent uptake seen in the vessel at the same level on the coronal image **(bottom right)**. The same plaque imaged at 6 weeks post angioplasty demonstrates dampened signal uptake **(far right, red arrow)**, with a patient who did not develop restenosis. Non-contrast CT **(top left)** with calcification of the vessel, ^18^F-FDG PET **(top right)** and fused ^18^F-NaF PET/CT **(bottom left)** of the superficial femoral artery **(blue arrow)** at the level mid-thigh, demonstrating significant vessel uptake in a patient with tissue loss in the left leg. In addition, there is prominent uptake seen across the whole vessel coronal image **(bottom right)**, in comparison with the contralateral leg. The same plaque imaged at 6 weeks post angioplasty demonstrates a persistent signal uptake **(far right)**, in a patient who did develop restenosis. CT = computed tomography; ^18^F-FDG = ^18^F-fluorodeoxyglucose; ^18^F-NaF = ^18^F-sodium fluoride; PET = positron emission tomography.

The overall median baseline ^18^F-FDG TBR_max_ was 1.79 (IQR: 1.60 to 2.29), with a median 6-week TBR_max_ of 1.21 (IQR: 1.07 to 2.34). The median baseline ^18^F-NaF TBR_max_ was 1.78 (IQR: 1.62 to 2.50), with a median 6-week TBR_max_ of 1.29 (IQR: 1.17 to 2.60). In general, there was a 32% decrease in ^18^F-FDG (p = 0.034) and a 28% decrease in ^18^F-NaF (p = 0.043) uptake from baseline to 6 weeks after the angioplasty. Lesion ^18^F-FDG and ^18^F-NaF uptake were highly correlated both at baseline (*r* = 0.818, p < 0.001) and at 6 weeks (*r* = 0.726, p < 0.001).

**Figure 2:**
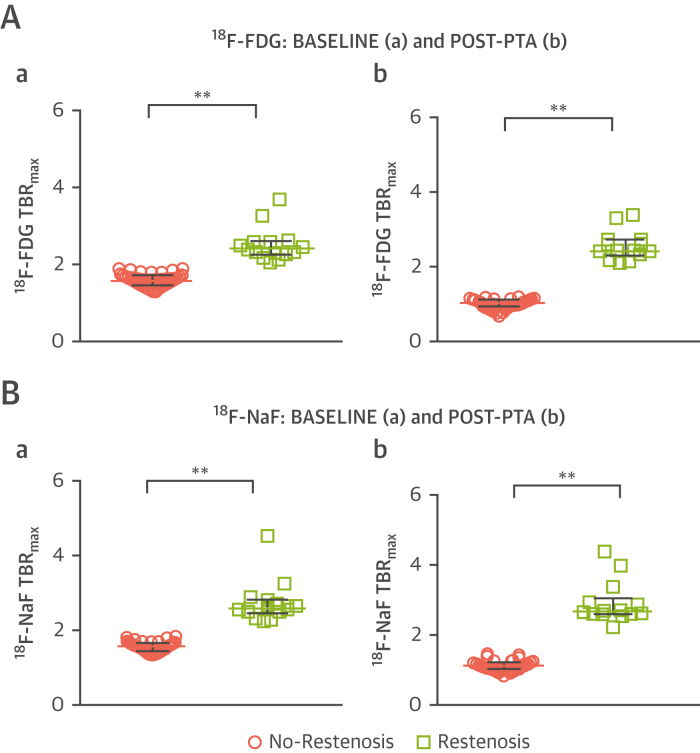
Comparison Between ^18^F-FDG and ^18^F-NaF Signal Stratified by Restenosis Status at 12 Months, for Both Baseline and Post-PTA Tukey dot plots to illustrate the comparison between ^18^F-FDG TBR_max_**(A)** at baseline imaging **(a)** and post-PTA **(b)** comparing patients who suffered from restenosis (n = 14, **green**) and those who did not (n = 26, **pink**). Mann-Whitney *U* test used for comparisons. Dots are actual median TBR_max_ values per patient, bars demonstrate median + interquartile range. **p < 0.001, *p <0.05. Tukey dot plots to illustrate the comparison between ^18^F-NaF TBR_max_**(B)** at baseline imaging **(a)** and post-PTA **(b)** comparing patients who suffered from restenosis (n = 14, **green**) and those who did not (n = 26, **pink**). Mann-Whitney *U* test used for comparisons. **Dots** are actual median TBR_max_ values per patient, **bars** demonstrate median + interquartile range. **p < 0.001, *p <0.05. TBR_max_ = target-to-background ratio maximum; other abbreviations as in Figure 1.

In patients who developed anatomic restenosis within 12 months, both baseline ^18^F-FDG TBR_max_ (1.63 [IQR: 1.52 to 1.78] vs. 2.43 [IQR: 2.29 to 2.61], p < 0.001) (Figure 2A) and ^18^F-NaF TBR_max_ (1.69 [IQR: 1.54 to 1.77] vs. 2.61 [IQR: 2.50 to 2.77], p < 0.001) (Figure 2B) were substantially higher than those without restenosis, and this increase persisted out to 6 weeks. In patients without restenosis, both ^18^F-FDG TBR_max_ (1.63 [IQR: 1.52 to 1.78] vs. 1.11 [IQR: 1.03 to 1.21], p = 0.034) and ^18^F-NaF TBR_max_ (1.69 [IQR: 1.55 to 1.77] vs. 1.19 [IQR: 1.10 to 1.29], p = 0.047) decreased by 6 weeks. In the contralateral (untreated, asymptomatic) limb, tracer levels for both ^18^F-FDG and ^18^F-NaF were comparable between the two timepoints, with no significant difference noted. The overall median baseline ^18^F-FDG TBR_max_ was 1.35 (IQR: 1.20 to 1.70), with a median 6-week TBR_max_ of 1.34 (IQR: 1.19 to 1.70). The median baseline ^18^F-NaF TBR_max_ was 1.54 (IQR: 1.36 to 1.72), with a median 6-week TBR_max_ of 1.55 (IQR: 1.36 to 1.71). In general, 6-week tracer levels were comparable with baseline levels (*r* = 0.973, p < 0.001).

### Clinical restenosis

All patients who had anatomic restenosis, re-presented with recurrence of symptoms, before the 12-month duplex scan. Symptom recurrence included stable claudication (n = 7), worsening claudication (n = 5), and worsening pain with non-healing ulcer (n = 2). In the no-restenosis group, 4 people developed symptoms during follow-up, which was manifest by a deterioration of walking distance.

**Figure 3:**
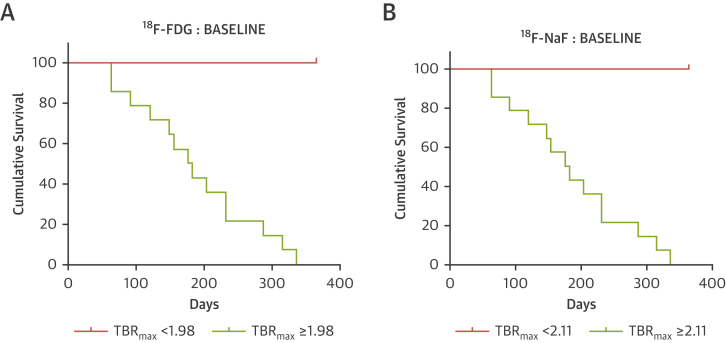
Restenosis Probability Kaplan-Meier curves for ^18^F-FDG **(A)** and ^18^F-NaF **(B)** signal at baseline categorized according to TBR_max._ Cut-off values derived from R function recursive partitioning and regression analyses. Log-rank test p < 0.001, censored data presented. Abbreviations as in Figures 1 and 2.

The predictive value of both ^18^F-FDG (with a cut-off TBR_max_ value of 1.98) and ^18^F-NaF (with a cut-off TBR_max_ value of 2.11) PET was highly discriminatory for the occurrence of restenosis at 1 year (p < 0.0001, log-rank p < 0.001) (Figure 3). In contrast, there was no difference in the index lesion calcium score between those who did or did not develop restenosis (1,143 AU [IQR: 435 to 2,144 AU] vs. 1,082 AU [IQR: 407 to 2,101 AU], respectively, p > 0.05). There was a significant positive correlation between symptomatic lesion ^18^F-NaF versus calcium score (*r* = 0.322, p = 0.043). No significant correlation was noted between ^18^F-FDG and calcium score.

## Discussion

In patients undergoing SFA angioplasty, we have demonstrated for the first time that baseline arterial inflammation and calcification activity can identify patients who will progress to symptomatic restenosis within a year. Moreover, symptomatic anatomic restenosis is associated with persistent vascular inflammation and calcification activity following PTA. This suggests that the atherosclerotic activity of the vascular wall determines downstream restenosis, and that this is a target for patient selection and potentially therapeutic manipulation.

This study is the first to evaluate both ^18^F-FDG and ^18^F-NaF uptake prospectively within a symptomatic PAD cohort. Interestingly, despite their disparate effects and pathways, we found that both tracers performed well in identifying patients who would develop restenosis within 12 months in all cases. Furthermore, in contrast to preceding PET imaging studies in the coronary and carotid vascular beds, we have demonstrated a high level of discrimination between both ^18^F-FDG and ^18^F-NaF arterial uptake with regard to restenosis status at 1 year, demonstrating a clear separation and no overlap of uptake values, a finding that in itself makes it very attractive for clinical decision-making. Interestingly, patients who did not develop restenosis demonstrated a significant decrease in tracer activity, which perhaps suggested a positive remodeling effect on plaque dynamics in these patients after angioplasty (Supplemental Figure 3). This may indicate that a subset of patients (who do not develop restenosis) observe a dampening of inflammation at the plaque level post-angioplasty, whereas patients who do develop restenosis do not respond in the same manner. It is unclear whether there is a phenotypic difference in plaque biology, and this warrants further investigation.

Restenosis following angioplasty is a burdensome and vexing problem, associated with poor outcomes, including mortality. PET/CT imaging in vascular disease is currently used as a research tool, without a clinical translational role. Given the increasing ease-of-access to PET imaging in many healthcare systems, and the simplicity and low cost of the radiotracers used, our study opens up the possibility of routine PET imaging in patients with PAD prior to intervention. Indeed, the cost of a single PET-CT examination is approximately £550 to £800 and re-intervention costs, in addition to those associated with the initial PTA, are £700 to £800 (22). However, a formal economic assessment of the use of PET in this role needs to be undertaken to determine its clinical effectiveness. This will also require external validation studies as well as clinical effectiveness trials where decisions to undertake PTA will be determined by baseline PET assessments.

In addition to guiding the initial therapeutic approach, peripheral vascular PET would be an ideal tool to assess the role of emerging endovascular therapies. The role of drug-coated balloon (DCB) angioplasty is not well-defined for PAD, predominately due to high costs and lack of data in complex populations (23). The use of PET imaging as a surrogate biomarker to assess the efficacy of DCB may aid in defining clear, objective validation of their use, on a plaque-specific basis. Furthermore, other novel local therapies including the Bullfrog Micro-Infusion Device (Mercator MedSystems) (24) or systemic therapies including PCSK9 inhibitors (25) and canakinumab could be assessed in this manner.

The mechanisms associated with vascular restenosis include endothelial denudation, oxidative stress, proliferating macrophages and vascular smooth muscle cells (26), and constrictive arterial wall remodeling (6). Many of these processes will be reflected in uptake of ^18^F-FDG and ^18^F-NaF. It is clear that ^18^F-FDG uptake is reflective of macrophage activity at the plaque-level, which has been implicated in restenosis pathophysiology (27). Previous studies have highlighted the role of calcification in restenosis (28) and our findings indicate that microcalcification (>50 μm) is associated with high-risk atherosclerotic plaques in both the coronary and carotid arteries 29, 30, 31. Moreover, it provides a marker of disease activity in other peripheral vascular conditions, such as abdominal aortic aneurysm disease, where it predicts aneurysm growth, and the risk of surgery or rupture (32).

### Study limitations

Patients with diabetes on insulin were excluded because elevated blood glucose levels preclude accurate ^18^F-FDG uptake measurements. The equivalence in performance between ^18^F-FDG and ^18^F-NaF in this study and the fact that ^18^F-NaF can be used in patients with diabetes without restrictions mean that ^18^F-NaF may be preferred for future studies aimed at predicting restenosis. The study was limited to patients with TASC A and B lesions to reduce variability and heterogeneity and because angioplasty is the recommended treatment for these lesions. Debate continues as to the optimal management (endovascular or open surgical) of more complex lesions 3, 33. Furthermore, this study did not look at patients who underwent SFA stenting, which has an increasing role in the lower limb. These data will require prospective external validation, and the generalizability of the current findings is limited by the modest sample size and single-center setting.

## Conclusions

Noninvasive molecular imaging using ^18^F-FDG and ^18^F-NaF PET of patients undergoing SFA angioplasty reliably identifies those who will develop restenosis at 1 year. Additionally, our data suggest that restenosis occurs because of incomplete resolution of inflammation and micro-calcification after angioplasty. Future studies are now needed to establish whether PET can improve the management of patients with symptomatic PAD by identifying those at risk of restenosis in a larger cohort, and to determine whether this imaging technique might be used as a platform to test anti-restenosis interventions.

Perspectives **COMPETENCY IN MEDICAL KNOWLEDGE:** Major determinants in restenosis following lower limb angioplasty have been based on systemic risk factors. To date, no independent factors of plaque biology have the ability to identify high-risk restenosis patients. PET with ^18^F-labelled FDG and NaF can provide plaque-specific data that conventional imaging does not provide.

**TRANSLATIONAL OUTLOOK 1:** Detection of levels of inflammation and microcalcification at the plaque level can allow for identification of the high-risk plaque that may experience restenosis after percutaneous revascularization.

**TRANSLATIONAL OUTLOOK 2:** Lower limb plaque imaging using PET/CT is reproducible and feasible in many healthcare systems and the simplicity and low cost of tracers used opens up the possibility of routine PET imaging in patients with PAD prior to intervention.
