# Supplementary material for: Vascular Positron Emission Tomography and Restenosis in Symptomatic Peripheral Arterial Disease: A Prospective Clinical Study
Source: JACC Cardiovasc Imaging. 2020 Apr;13(4):1008–17. doi: 10.1016/j.jcmg.2019.03.031 (PMC7136751; doi:10.1016/j.jcmg.2019.03.031)
Supplement: Supplemental Data [file mmc1.docx]

**Vascular Positron Emission Tomography and Restenosis in Symptomatic Peripheral Arterial Disease: A Prospective Clinical Study**

^a,b^Mohammed M. Chowdhury MB ChB MRCS; ^b^Jason M. Tarkin, MBBS MRCP PhD; ^c^Mazen S. Albaghdadi MD MSc; ^f^Nicholas R. Evans MB BChir MRCP PhD; ^b^Elizabeth P.V. Le BA; ^d^Thomas B. Berrett PhD; ^a^Umar Sadat PhD FRCS; ^e^Francis R. Joshi, PhD MRCP; ^f^Elizabeth A. Warburton, DM MRCP; ^g^John R. Buscombe, MD FRCP; ^a^Paul D. Hayes, MD FRCS; ^h^Marc R. Dweck, MD PhD, FACC; ^h^David E. Newby, MD, PhD; ^b^James H.F. Rudd, PhD, FRCP, FESC†; ^a^Patrick A. Coughlin, MD FRCS†

^a^ Division of Vascular Surgery, Department of Surgery, Addenbrooke’s Hospital, University of Cambridge, UK;

^b^ Department of Cardiovascular Medicine, Addenbrooke’s Hospital, University of Cambridge, UK;

^c^ Cardiovascular Research Center, Division of Cardiology, Massachusetts General Hospital, Harvard Medical School, Boston, Massachusetts, USA;

^d^ Statistical Laboratory, Department of Pure Mathematics and Mathematical Sciences, University of Cambridge, UK;

^e^ Heart Center, Rigshospitalet, Denmark;

^f^ Department of Clinical Neurosciences, University of Cambridge, UK;

^g^ Department of Nuclear Medicine, Addenbrooke’s Hospital, University of Cambridge UK;

^h^ British Heart Foundation for Cardiovascular Science, University of Edinburgh, Edinburgh, United Kingdom

† Joint senior authors – J.H.F.R., and P.A.C

**BRIEF TITLE:** PET/CT Identifies Restenosis in PAD Patients

**DISCLOSURES:** None

**FUNDING:** This study, and MMC was funded by Fellowships from the Royal College of Surgeons of England and the British Heart Foundation (BHF; FS/16/29/31957). JMT is supported by a Wellcome Trust research training fellowship (104492/Z/14/Z). NRE is supported by a fellowship The Dunhill Medical Trust (RTF44/0114). TBB is supported by ESPRC programme grant StatScale (EP/N031938/1). JHFR is supported by the NIHR, BHF, Wellcome Trust, and HEFCE. MRD is supported by the British Heart Foundation (FS/14/78/31020) and is the recipient of Sir Jules Thorn Award for Biomedical Research (15/JTA). DEN is supported by the British Heart Foundation (CH/09/002, RE/13/3/30183, RM/13/2/30158) and is the recipient of a Wellcome Trust Senior Investigator Award (WT103782AIA). This work was supported by the NIHR Cambridge Biomedical Research Centre and the Cambridge Clinical Trials Unit.

**ADDRESS FOR CORRESPONDENCE**

Mr. Mohammed M. Chowdhury

Divisions of Vascular Surgery and Cardiovascular Medicine, University of Cambridge

Box 212, Addenbrooke’s Cambridge University Hospital,

Hills Road, Cambridge, UK, CB2 2QQ

Email : mmc59@cam.ac.uk

Tel : +44 (0) 7793 028033

Fax: +44 (0) 1223 331505

**ACKNOWLEDGEMENTS**

We gratefully acknowledge the volunteers who participated in this study, and assistance provided by the radiographers and staff working in the PET/CT department of Addenbrooke’s Hospital. We also thank the vascular studies unit team at Addenbrooke’s hospital, including chief vascular scientists Robert Elliot and his team, Edmund Ramage, Matthew Slater, Charlotte Taylor, and Laura Scott, for their assistance with arterial duplex imaging.

**SUPPLEMENTARY MATERIAL**

**METHODS**

**Study design**

The Calcification and Inflammation in Restenosis injury in Lower limb arterial disease post-Angioplasty (CIRLA) study was a single-centre, prospective imaging study. This study is registered with ISRCTN.com (ISRCTN34690731).

**Patients**

Patient demographics, medical history and medications were determined from the hospital electronic record system and interview. Ischaemic heart disease was defined as a clinical diagnosis of angina, a prescription of anti-anginal drugs or a history of myocardial infarction or a coronary revascularization procedure. Cerebrovascular disease / transient ischaemic attack was determined as a previous history of a cerebral event with associated neurology. Chronic kidney disease was defined as an estimated glomerular filtration rate ≤ 59 ml/min/1.73m^2^).

**Clinical Imaging**

Visit one: patients underwent ^18^F-NaF PET/CT scan and CT angiogram of the SFA. Prior to PET imaging, a scouting and non-contrast CT (120kV, 10mA, 180º) was performed for co-registration and attenuation correction with the participant’s legs immobilized with a loosely applied tourniquet at the ankles. After intravenous injection of ^18^F-NaF, there was a 60-minute circulation time before static PET imaging. The scan extended from the iliac crest to the tibial plateau to ensure the entire superficial femoral artery was imaged. CT parameters were 120kV with a pitch of 1.375. The reconstruction was at 3.75mm slices, at 3.27mm intervals, with a field of view of 50cm. PET parameters were 4 minutes acquisition per bed position with the first reconstruction in VPFX (time-of-flight), and an attenuated corrected field of view of 70cm. CT angiography: This scan was performed at the end of the PET acquisition, on the same scanner. The scan used helical acquisition with a kV of 120, mA of max 400, with a field of view of 350-380mm yielding a typical spatial resolution of 0.7x0.4x5.0mm^3^.

Visit two: PET-CT imaging was repeated using the alternate tracer (^18^F-FDG), with at least 24 hours between PET imaging sessions to allow full radiotracer decay. Imaging protocol was the same as above, but no CT angiogram was performed. Patients were instructed to fast for six hours prior to ^18^F-FDG imaging, with capillary blood glucose monitored to ensure this level was <7.0mmol/L prior to scanning, as per usual “real-world” clinical imaging protocols. Based on previous work^1^, a 90-minute circulation time was used for ^18^F-FDG imaging.

Visit three: ^18^F-NaF PET/CT was repeated 6 weeks after angioplasty using the same technique as the pre-angioplasty scan; Visit four: ^18^F-FDG PET/CT was repeated 6 weeks after angioplasty using the same technique as the pre-angioplasty scan.

The total mean injected dose of ^18^F-NaF was 401.4MBq (399.68-406.03) and ^18^F-FDG 595.1MBq (584.53-611.13). The maximum total estimated radiation exposure per patient was 24mSv.

**Statistical analysis, reproducibility, and power calculation**

Published literature suggests that approximately 50% of patients will develop restenosis within 12 months after PTA^2^. ^18^F-NaF was used as the primary PET tracer of interest given the suggested role of calcification in restenosis. In collaboration with the Cambridge Clinical Trials Unit and based on our initial data, it was estimated that there would be a difference of 0.4 in the ^18^F-NaF TBR_max_ between patients who develop and those who do not develop restenosis. Pilot data from our previous work demonstrated a TBR_max_ of 1.94 with a standard deviation of 0.29 for ^18^F-NaF uptake, and TBR of 1.86 with a standard deviation of 0.89 for ^18^F-FDG uptake. Based on this, with 80% power and 5% alpha, it was estimated that 38 patients would be required to detect a difference of 0.4 in the TBR_max_ for ^18^F-NaF between groups. To allow for 20% dropout rate – we aimed to have 46 patients make up the initial study cohort.

**Justification of statistical approach**

The data analysed was, in most cases, perfectly separated by the PET scores, in that, for example, all patients with an ^18^F-FDG baseline (B) score of >1.98 had restenosis, while all patients with an ^18^F-FDG baseline (B) score of <1.98 did not. This is illustrated using classification trees. For variables leading to perfect separation it is not possible to fit a logistic regression model in the standard approach given the maximum likelihood estimator of the parameters of this model does not exist. The likelihood can in fact be made arbitrarily large through larger and larger choices of the parameters.

In order to provide statistical conclusions on the effect of the PET variables on the probability of a patient having restenosis, therefore, we could not use standard logistic regression, and instead used the Kolmogorov-Smirnoff test. The null hypothesis here was that the distribution of the PET score in question was the same for patients with or without restenosis. For all the scores exhibiting perfect separation the p-value was 8.618 x 10^-11^, while for the two scores not exhibiting perfect separation the p-value was 3.529 x 10^-5^. We may conclude from these tests that all of the PET scores have a significant effect on the probability of a patient having restenosis.

**RESULTS**

Excellent inter-observer reliability was reproduced for analysis of PET data. Bland-Altman analysis demonstrated a low bias of 0.04 ± 0.40 for FDG reads and similarly for NaF reads (-0.02 ± 0.40), with an associated intra-class correlation coefficient of 0.96 [95% CI: 0.94-0.97]; and 0.94 95% CI: 0.93-0.96], respectively (Figure S2).

**REFERENCES**

1 Bucerius J, Mani V, Moncrieff C, *et al.* Optimizing 18F-FDG PET/CT imaging of vessel wall inflammation: the impact of 18F-FDG circulation time, injected dose, uptake parameters, and fasting blood glucose levels. *Eur J Nucl Med Mol Imaging* 2014; **41**: 369–83.

2 Amighi J, Schillinger M, Dick P, *et al.* De novo superficial femoropopliteal artery lesions: peripheral cutting balloon angioplasty and restenosis rates--randomized controlled trial. *Radiology* 2008; **247**: 267–72.

3 Tawakol A, Fayad Z, Mogg R. Intensification of Statin Therapy Results in a Rapid Reduction in Atherosclerotic Inflammation: Results of A Multi-Center FDG-PET/CT Feasibility Study. *J Am …* 2013; **62**: 2013.

**SUPPLEMENTARY FIGURES**


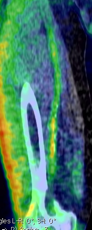

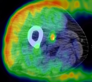


Measured ROI Values (background corrected)

Mean TBR

Max TBR

1.4

1.6

1.3

1.5

2.3

2.6

2.2

2.4

2.3

2.5

2.4

2.6

2.1

2.5

SFA lesion subject to angioplasty (index lesion)

2.6

2.4

2.5

2.6

2.5

Symptomatic SFA TBR_max_ = 2.52

Coronal Plane

Axial Plane

Merged PET-CT


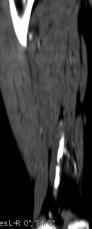


CT angiogram

Proximal

SFA

**Figure S1. PET/CT Vascular Reads – Index Lesion**

Regions of interest were drawn around the SFA (in axial orientation) to provide a maximum standardised uptake value (SUV) for each region of interest. Index lesions, identified by duplex and computed tomography (demonstrated on the coronal plane CT angiogram, in green), were then analysed constructing a stack of diseased ROIs. SFA; superficial femoral artery, ROI; region-of-interest, TBR; target-to-background ratio, CT; computed tomography; PET, positron emission tomography. Adapted from reference^3^


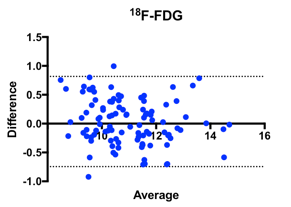

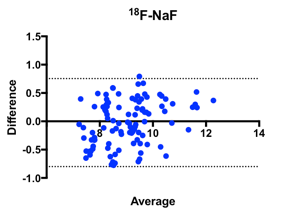


**Figure S2. Bland – Altman Analysis of inter-observer reliability**

^18^F-FDG reads for 3mm slices within the SFA (n=100), with a low bias of 0.04 ± 0.40, and (b) ^18^F-NaF comparison for 3mm slice reads within the SFA (n=100), with a low bias of -0.02 ± 0.40 (associated intra-class correlation coefficient of 0.96 [95% CI: 0.94-0.97]; and 0.94 95% CI: 0.93-0.96], respectively).


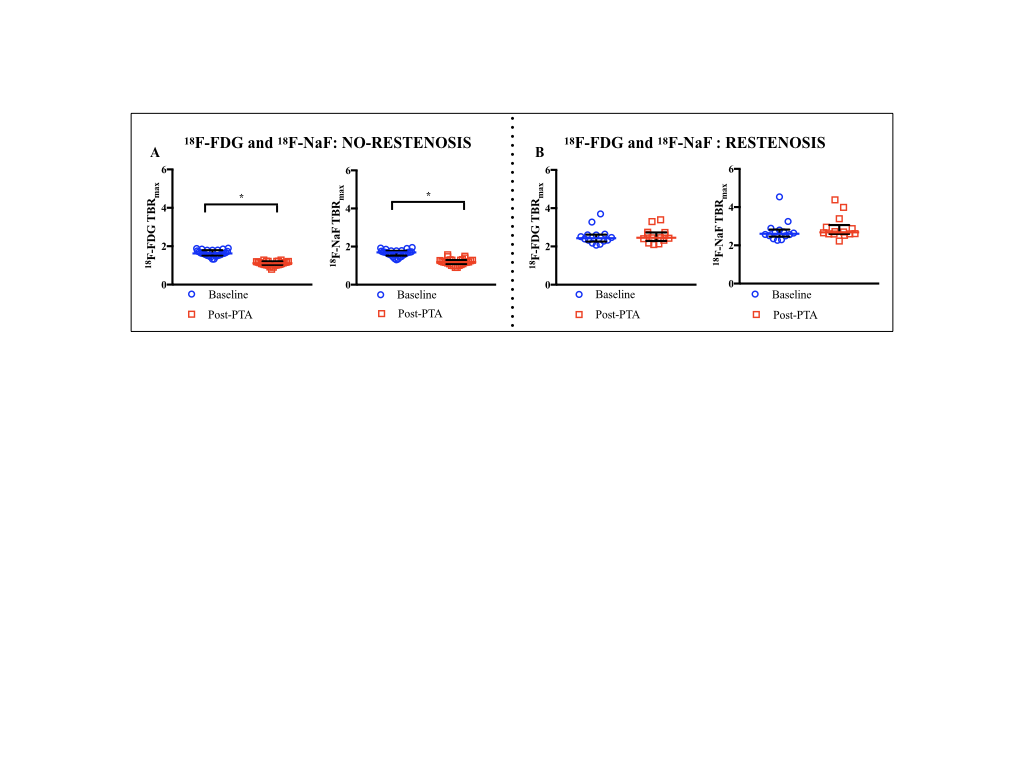


**Figure S3. Comparison between ^18^F-FDG and ^18^F-NaF signal at baseline versus post-PTA stratified by no-restenosis group (A), and restenosis group (B)**

Tukey dot plots to illustrate the comparison between baseline and 6-week post-angioplasty PET reads in the no-restenosis group with ^18^F-FDG and ^18^F-NaF (A) and in the restenosis group with ^18^F-FDG and ^18^F-NaF (B). Mann-Whitney U test interrogation revealing strong statistical difference between the two groups. ^18^F-FDG; ^18^F-fluorodeoxyglucose, ^18^F-NaF; ^18^F-sodium fluoride, TBR_max_; target-to-background ratio maximum, SFA; superficial femoral artery, dots are actual median TBR_max_ values per patient, bars demonstrate median + interquartile range. ** p <0.001, * p <0.05

| **Patient baseline characteristics of study cohort (n=50)** | | | |
| --- | --- | --- | --- |
| Age, median (IQR) | |  | 70 (65-78) |
| Men, n (%) |  | | 33 (66%) |
| **Sub-group, n (%)** |  | |  |
|  | Intermittent claudication | | 41 (82%) |
|  | Critical limb ischaemia | | 9 (18%) |
|  | TASC A | | 32 (64%) |
|  | TASC B | | 18 (36%) |
| **Previous Medical History, n (%)** | | | |
|  | Hypertension | | 39 (78%) |
|  | Non-insulin dependent diabetes | | 17 (34%) |
|  | Ischaemic heart disease / MI | | 19 (38%) |
|  | Cerebrovascular event / TIA | | 5 (10%) |
| Smoker, n (%) |  | | 5 (10%) |
| Ex-smoker, n (%) |  | | 36 (72%) |
| **Medication, n (%)** |  | |  |
|  | Clopidogrel | | 10 (20%) |
|  | Aspirin | | 34 (68%) |
|  | Anticoagulation | | 4 (8%) |
|  | Dyprimadole | | 1 (2%) |
|  | Statin | | 45 (90%) |
|  | ACE-inhibitor | | 27 (54%) |
| BMI (kg/m^2^), mean ± SD | | | 28·38 ± 4·67 |
| ABPI |  | | 0·71 (0·68-0·79) |
| **Lipid Profile, mean ± SD** | | | |
|  | Total cholesterol | | 4·33 ± 1·01 |
|  | HDL cholesterol | | 1·31 ± 0·39 |
|  | LDL cholesterol | | 2·12 ± 0·80 |
|  | Triglycerides | | 1·99 ± 0·92 |
|  | HDL:Chol Ratio | | 3·52 ± 1·10 |
| **Limited Bone and Renal Profile, median (IQR)** | | |  |
|  | Creatinine (µmol/L) | | 78·5 (66·75-89) |
|  | Corrected calcium (mmol/L) | | 2·36 (2·31-2·43) |
| High sensitivity CRP (mg/dL), median (IQR) | | | 2·53 (0·92-8·33) |

**Table S1. Clinical Characteristics of Patients**

N=50. Values reported as whole numbers, percentages in parentheses. Non-parametric data presented as median + interquartile range, and normally distributed data as mean ± standard deviation. HDL; high density lipoprotein, LDL; low density lipoprotein, eGFR; estimated glomerular filtration rate, CRP; C-reactive protein, ABPI; ankle-brachial pressure index, ACE; angiotensin-converting enzyme, MI; myocardial infarction, TASC; TransAtlantic Inter-Society Consensus, TIA; transient ischemic attack.
